# Perioperative use of renin-angiotensin system inhibitors and outcomes in patients undergoing cardiac surgery

**DOI:** 10.1038/s41467-019-11678-9

**Published:** 2019-09-13

**Authors:** Qian Ding, Zugui Zhang, Hong Liu, Huang Nie, Mark Berguson, Jordan E. Goldhammer, Nilas Young, Douglas Boyd, Rohinton Morris, Jianzhong Sun

**Affiliations:** 1 Department of Anesthesiology, Thomas Jefferson University, Philadelphia, PA 19107 USA; 2 Department of anesthesiology, Tangdu Hospital, Xian, PR China; 3 The Value Institute, Christiana Care Health System, Newark, DE USA; 4 Department of Anesthesiology and Pain Medicine, University of California Davis Medical Center, Sacramento, CA 95817 USA; 5 Department of anesthesiology, Xijing Hospital, Xian, PR China; 6 Division of Cardiothoracic Surgery, University of California Davis Medical Center, Sacramento, CA 95817 USA; 7 Division of Cardiothoracic Surgery, Sidney Kimmel Medical College, Thomas Jefferson University, Philadelphia, PA 19107 USA

**Keywords:** Therapeutics, Interventional cardiology

## Abstract

It remains disputable about perioperative use of renin-angiotensin system inhibitors (RASi) and their outcome effects. This multicenter retrospective cohort study examines association between use of perioperative RASi and outcomes in patients undergoing coronary artery bypass graft and/or valve surgery. After the exclusion, the patients are divided into 2 groups with or without preoperative RASi (PreRASi, n = 8581), or 2 groups with or without postoperative RASi (PostRASi, n = 8130). With using of propensity scores matching to reduce treatment selection bias, the study shows that PreRASi is associated with a significant reduction in postoperative 30-day mortality compared with without one (3.41% vs. 5.02%); PostRASi is associated with reduced long-term mortality rate compared with without one (6.62% vs. 7.70% at 2-year; 17.09% vs. 19.95% at 6-year). The results suggest that perioperative use of RASi has a significant benefit for the postoperative and long-term survival among patients undergoing cardiac surgery.

## Abstract (web summary)

Renin-angiotensin system inhibitors (RASi) are beneficial in several classes of cardiovascular patients. However, whether their perioperative use is beneficial and/or safe in cardiac surgery is unclear. Here the authors perform a multicenter retrospective cohort study showing that preoperative and postoperative use of RASi is associated with reduction of mortality in patients undergoing cardiac surgery.

## Introduction

In the early 2000s, two large-randomized clinical trials, the Heart Outcomes Prevention Evaluation (HOPE) trial^1^ and the European Trial on Reduction of Cardiac Events with Perindopril in Stable Coronary Artery Disease^2^, showed that in patients with risk of cardiovascular disease (CVD) but without heart failure, angiotensin converting enzyme inhibitors (ACE inhibitors) resulted in a relative risk (RR) reduction of about 20% in mortality, MI, and stroke in 4–5 years follow-up. The effects were independent of age, gender, concomitant diseases, or therapy. Thus far, multiple clinical trials and cohort studies have demonstrated that ACE inhibitors are beneficial to patients with hypertension, diabetes, renal disease, heart dysfunction, also to patients with coronary artery disease (CAD), and valve diseases^1–8^.

Meanwhile, despite accumulating evidence that ACE inhibitors are effective drugs in a broad range of patients with risk of CVD and increasing numbers of patients are treated with ACE inhibitors prior to cardiac surgery, whether or not ACE inhibitors should be continued or given perioperatively remains controversial. Results from previous clinical studies are conflicting and raise more questions than answers^9–13^. The Guidelines (2011) for coronary artery bypass graft (CABG) from the American Heart Association and American College of Cardiology stated that it is uncertain about the safety of the preoperative administration of ACE inhibitors or angiotensin II receptor blockers (ARBs) in patients on chronic therapy and the safety of initiating ACE inhibitors or ARBs before hospital discharge^14^. In the updated 2014 ESC/EACTS Guidelines, they state that ACE inhibitors might be stopped 1–2 days before CABG to avoid the potential deleterious consequences of perioperative hypotension^15^. Thus, there is a need to investigate the safety and effectiveness of preoperative and postoperative RASi therapy in large cardiovascular cohorts.

In this study, we examined both preoperative and postoperative (or perioperative) use of RASi, including ACE inhibitors, ARBs, and direct renin inhibitors, on outcomes of patients undergoing CABG and/or valve surgery. The results show that perioperative use of RASi has a significant benefit for the postoperative and long-term survival among patients undergoing CABG and/or valve surgery.

## Results

### Characteristics of the study patients

**Fig. 1:**
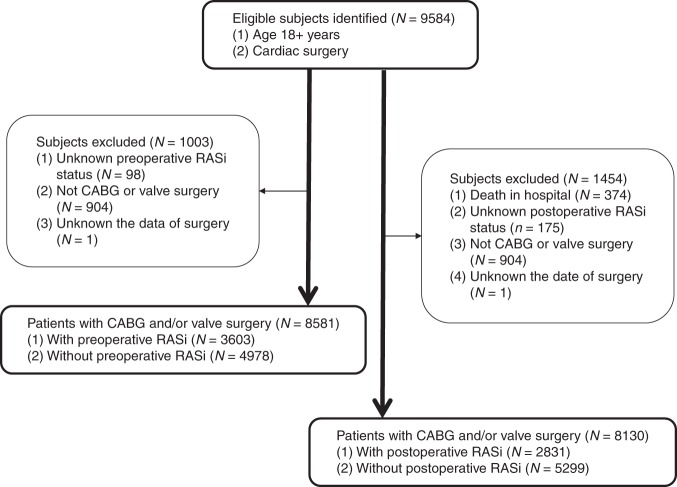
Study population recruitment summary. RASi renin-angiotensin system inhibitors, CABG coronary artery bypass graft

Overall 8581 patients with CABG and/or valve surgery were included in preoperative RASi (PreRASi) groups of this study. Among them, 3603 patients (42.0%) received PreRASi, and 4978 (58.0%) did not (no-PreRASi). Postoperatively (*n* = 8130), 2831 patients (34.8%) were prescribed RASi at discharge (PostRASi), and 5299 patients (65.2%) did not (no-PostRASi) (Fig. 1). Baseline demographic and clinical data of the patients were presented in Table 1. Before adjustment using propensity scores matching (PSM) or inverse probability weighted (IPW), more patients taking preoperative or postoperative RASi than not taking RASi had diabetes, hypertension, angina, congestive heart failure, and previous myocardial infarction. A greater number of RASi-treated patients were receiving other medications, including beta-blockers, aspirin, and lipid-lowering drugs. Patients taking PreRASi had higher BMI and increased incidence of cerebrovascular disease, peripheral vascular disease, a few of minutes longer of cardiopulmonary bypass time and cross clamp time, a slightly more using artery grafts and off-pump, more using PostRASi as well as decreased incidence of chronic lung disease, and urgent CABG or valve surgery. For patients taking PostRASi, there were similar changes as the patients with PreRASi, but decreased incidence of smoking and more with PreRASi and discharge aspirin (Table 2). After adjustment with PSM, all of clinical covariates were well balanced and no significant differences were found between the two groups except for using RASi.

**Table 1:** Demographic and clinical characteristics: preoperative RAS inhibitors

	Unadjusted			Inverse probability weighted adjusted			Matched data		
	PreRASi (*n* = 3603)	No-PreRASiI (*n* = 4978)	*p*-value	PreRASi (*n* = 3603)	No-PreRASi (*n* = 4978)	*p*-value	PreRASi (*n* = 3284)	No-PreRASi (*n* = 3284)	*p*-value
Age	65.9 ± 11.7	64.8 ± 13.6	<0.0001	65.6 ± 18.9	65.3 ± 17.2	0.34	65.9 ± 11.8	66.0 ± 12.4	0.70
Male	68.9	67.4	0.14	68.2	68.0	0.83	68.4	68.7	0.83
BMI	29.2 ± 6.2	28.6 ± 6.2	<0.0001	28.9 ± 9.5	28.9 ± 8.3	0.74	29.1 ± 6.1	29.1 ± 6.3	0.88
Diabetes	38.7	27.6	<0.0001	32.9	32.5	0.60	35.8	35.2	0.66
Smoker	40.7	39.5	0.26	40.3	40.1	0.72	40.2	40.4	0.92
Hypertension	87.3	69.9	<0.0001	78.4	77.4	0.14	86.2	87.0	0.35
Cerebrovascular disease	18.1	15.4	0.001	16.8	16.7	0.83	17.5	17.1	0.70
Peripheral arterial disease	13.9	11.6	0.002	12.7	12.6	0.91	13.4	12.8	0.49
Chronic lung disease	18.8	20.1	0.04	20.3	19.8	0.49	19.6	19.7	0.90
Family history CAD	40.5	38.3	0.04	39.7	39.3	0.58	40.6	41.3	0.58
Angina	37.2	34.6	0.02	36.1	35.9	0.73	37.8	38.3	0.51
Congestive heart failure	32.7	28.6	<0.0001	31.4	30.7	0.30	30.7	30.5	0.89
Previous MI	36.4	30.2	<0.0001	33.5	33.0	0.47	35.0	35.1	0.94
Beta blockers	72.6	63.3	<0.0001	68.9	67.6	0.09	71.4	71.5	0.87
Aspirin	75.9	66.6	<0.0001	71.3	70.7	0.34	74.5	74.5	0.98
Lipid lowering	82.2	74.1	<0.0001	78.3	77.7	0.35	81.1	81.5	0.73
Urgent	49.9	52.9	0.01	51.7	51.6	0.86	51.1	51.9	0.52
CABG	49.5	51.9	<0.0001	55.3	55.0	0.76	58.3	58.5	0.84
Valve	24.0	29.3	<0.0001	27.0	27.1	0.90	24.8	24.9	0.93
Post RAS inhibitor	46.0	25.6	<0.0001	45.8	26.7	<0.0001	46.0	28.5	<0.0001
Cardiopulmonary bypass time	130.7 ± 80.5	126.3 ± 79.6	0.012	128.2 ± 121.1	128.3 ± 107.0	0.96	127.5 ± 77.8	126.3 ± 79.7	0.52
Cross clamp time	93.8 ± 59.5	90.8 ± 58.9	0.020	92.3 ± 90.2	92.2 ± 79.3	0.97	91.8 ± 58.1	90.9 ± 58.9	0.53
Number of artery grafts			0.012			0.80			0.14
None	45.7	48.8		46.7	47.2		46.2	48.2	
1	45.5	42.4		44.4	44.0		45.3	42.8	
2 or more	8.8	8.8		8.9	8.8		8.5	8.9	
On-off pump			0.0021			0.80			0.39
Off pump	6.5	6.7		6.4	6.5		6.7	6.2	
On pump (full)	93.5	93.3		93.6	93.5		93.3	93.8	

Values are mean ± SD or %. C-index = 0.74. *p*-values are obtained via analyses using Pearson chi-square test or Wilcoxon rank-sum test (unadjusted) and then IPW or PSM (adjusted)

*BMI* body mass index, *MI* myocardial infarction

**Table 2:** Demographic and Clinical Characteristics: Post RAS Inhibitors

	Unadjusted			Inverse probability weighted adjusted			Matched	Data	
	PostRASi (*n* = 2831)	No-PostRASiI (*n* = 5299)	*p*-value	PostRASi (*n* = 2831)	No-PostRASi (*n* = 5299)	*p*-value	PostRASi (*n* = 2673)	No-PostRASi (*n* = 2673)	*p*-value
Age	65.8 ± 11.9	64.7 ± 13.2	0.0004	65.2 ± 20.5	65.1 ± 15.4	0.57	65.7 ± 12	65.8 ± 12.3	0.86
Male	68.4	68.6	0.82	68.7	68.5	0.63	68.4	68.4	0.98
BMI	29.1 ± 6.2	28.8 ± 6.2	0.09	28.9 ± 10.2	28.4 ± 7.5	0.64	29.1 ± 6.2	29.0 ± 6.2	0.61
Diabetes	37.8	29.5	<0.0001	32.7	32.5	0.64	36.2	35.9	0.80
Smoker	38.7	41.3	0.02	39.8	40.3	0.67	39.5	39.7	0.85
Hypertension	83.7	73.9	<0.0001	78.2	78.5	0.42	83.0	83.7	0.49
Cerebrovascular disease	16.8	16.0	0.34	16.1	16.7	0.53	17.0	16.3	0.53
Peripheral arterial disease	13.1	11.6	0.06	12.1	12.3	0.75	12.8	11.9	0.30
Chronic lung disease	18.5	19.5	0.30	19.40	19.0	0.82	18.8	18.7	0.94
Family history CAD	39.1	39.5	0.68	39.0	38.8	0.61	39.3	40.3	0.78
Angina	39.3	34.1	<0.0001	35.1	35.8	0.72	38.5	38.8	0.78
Congestive heart failure	32.3	27.7	0.0001	30.4	30.5	0.86	31.3	30.7	0.62
Previous MI	36.5	30.2	<0.0001	32.1	33.0	0.81	35.4	35.8	0.75
Discharge beta blockers	83.1	82.1	0.07	83.1	82.9	0.32	83.2	83.9	0.61
Discharge aspirin	85.7	84.2	0.03	85.7	85.8	0.69	86.8	85.1	0.25
Discharge lipid lowering	81.0	79.5	0.07	80.3	80.1	0.46	81.9	89.7	0.74
Discharge warfarin	24.5	22.8	0.07	24.0	23.5	0.35	24.1	22.0	0.15
Urgent	53.9	49.3	0.001	51.9	50.7	0.61	52.8	53.1	0.83
CABG	58.5	54.6	0.001	56.2	55.9	0.36	58.0	59.1	0.42
Valve	25.6	27.1	0.13	26.2	26.7	0.59	25.9	25.4	0.68
Pre RAS inhibitor	56.9	34.7	<0.0001	42.9	42.2	0.87	54.6	54.1	0.70

C-index = 0.66, values are mean ± SD or %. *p*-values are obtained via analyses using Pearson chi-square test or Wilcoxon rank-sum test (unadjusted) and then IPW or PSM (adjusted)

*BMI* body mass index, *MI* myocardial infarction, *CAD* coronary artery disease

**Fig. 2:**
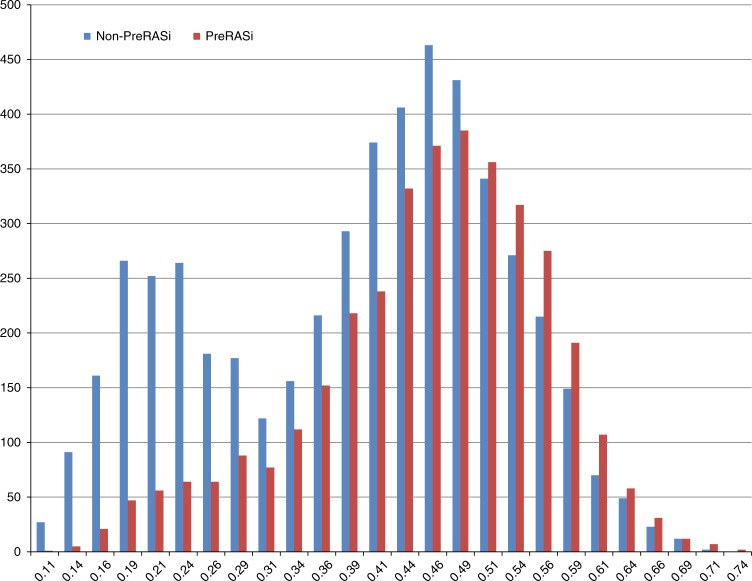
Distribution of propensity scores for preoperative RASi (PreRASi) in the patient populations (y-axis). The propensity score (x-axis) for PreRASi is the probability given baseline variables that any patient in either group would be selected for PreRASi

As expected, patients who took PreRASi had a lower probability of being selected as not taking RASi (No-PreRASi, Fig. 2), with the median and interquartile range of the propensity scores for PreRASi reflecting this difference (PreRASi group: median, 0.47; interquartile range, 0.40–0.53; no-PreRASi group: median, 0.42; interquartile range, 0.27–0.49).

### Effects of PreRASi on outcomes

Postoperatively, compared with no-PreRASi, PreRASi did not show a significant reduction in heart, brain, and renal complication. MACE, a composite outcome, did show a significant reduction with unadjusted analysis (*P* = 0.04), which became nonsignificant with the PSM analysis (*P* = 0.19). In contrast, compared with no-PreRASi, PreRASi was associated with a significant reduction in postoperative 30-day mortality (3.55% vs. 5.04%, OR 0.69, 95% CI 0.54–0.90, *P* =  0.01 with PSM or unadjusted analysis) (Table 3).

**Table 3:** Preoperative RAS inhibitors and postoperative outcomes

Outcome *n* (%)	Unadjusted				IPW adjusted		One-to-one matched			
	RASi (*N* = 3603)	Non RASi (*N* = 4978)	OR 95% CI	*p*-value	OR 95% CI	*p*-value	RASi (*N* = 3284)	Non RASi (*N* = 3284)	OR 95% CI	*p*-value
MACE	417 (11.6)	650 (13.1)	0.87 (0.76–0.99)	0.04	0.89 (0.81–0.97)	0.01	377 (11.48)	419 (12.76)	0.90 (0.77–1.05)	0.19
Perioperative MI	29 (0.80)	39 (0.78)	1.03 (0.63–1.67)	0.91	1.07 (0.76–1.48)	0.69	26 (0.79)	25 (0.76)	1.02 (0.58–1.78)	0.95
Heart block	51 (1.42)	58 (1.17)	1.22 (0.83–1.78)	0.31	1.50 (1.16–1.94)	0.01	48 (1.46)	40 (1.22)	1.19 (0.77–1.82)	0.42
Cardiac arrest	86 (2.39)	113 (2.27)	1.05 (0.79–1.40)	0.72	1.11 (0.91–1.41)	0.29	80 (2.44)	67 (2.04)	1.28 (0.92–1.79)	0.47
Permanent stroke	68 (1.89)	111 (2.23)	0.84 (0.62–1.14)	0.27	0.85 (0.69–1.01)	0.13	59 (1.80)	68 (2.07)	0.88 (0.62–1.25)	0.47
TIA	15 (0.42)	24 (0.48)	0.86 (0.45–1.65)	0.66	0.90 (0.57–1.43)	0.66	12 (0.37)	13 (0.40)	0.91 (0.41–2.02)	0.77
Coma	26 (0.72)	40 (0.80)	0.90 (0.55–1.47)	0.67	0.88 (0.62–1.25)	0.63	21 (0.64)	21 (0.64)	1.09 (0.59–2.03)	0.99
Renal failure	167 (4.64)	258 (5.18)	0.89 (0.73–1.09)	0.25	0.86 (0.75–0.97)	0.03	148 (4.51)	168 (5.12)	0.91 (0.72–1.15)	0.42
Total hrs ICU mean ± SD	112.4 ± 186	116.7 ± 179	4.51 (−3.27–12.3)	0.26	−1.94 (−10.4–6.2)	0.64	112.1 ± 188	112.6 ± 167	0.55 ± 178	0.90
Readmission	502 (13.93)	624 (12.54)	1.13 (0.996–1.28)	0.06	1.10 (1.01–1.21)	0.02	467 (14.22)	428 (13.03)	1.10 (0.95–1.27)	0.20
30-day mortality	128 (3.55)	251 (5.04)	0.69 (0.54–0.85)	0.01	0.67 (0.58–0.76)	<0.0001	112 (3.41)	165 (5.02)	0.69 (0.54–0.90)	0.01

Values are mean ± SD or number or %. *p*-values are obtained via analyses using Pearson chi-square test or Wilcoxon rank-sum test (unadjusted) and then IPW or PSM (adjusted)

*MACE* major adverse cardiovascular events, *MI* myocardial infarction, *TIA* transient ischemic attack

**Fig. 3:**
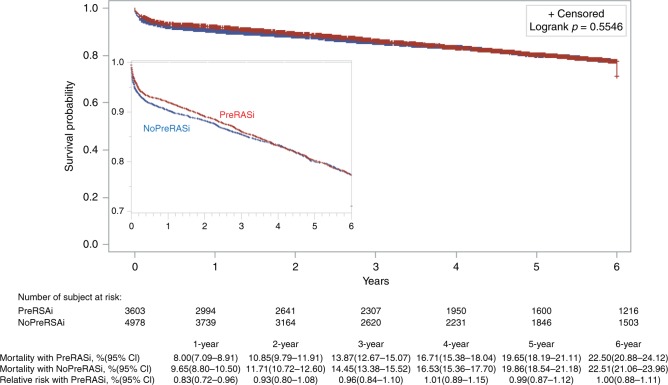
Rates of survival in patients. Cumulative mortality with and without preoperative RASi (PreRASi) and the relative risk (95% confidence intervals in brackets) of PreRASi as compared with no-PreRASi are shown*. Logrank p*-value is analyzed with using the Kaplan–Meier method (unadjusted). The inset shows the same data on an enlarged *y* axis

**Fig. 4:**
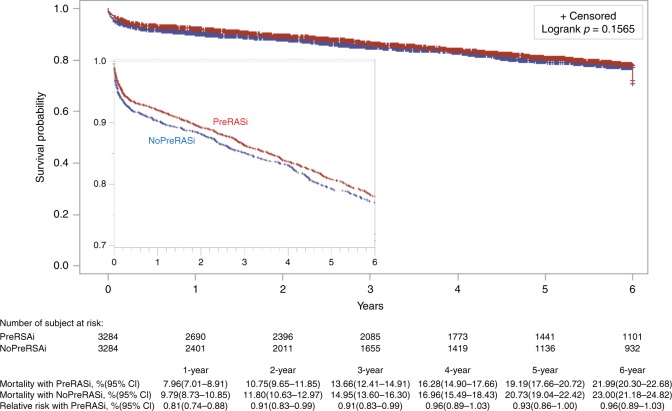
Rates of survival in patients. Cumulative mortality with and without preoperative RASi (PreRASi) and the relative risk (95% confidence intervals in brackets) of PreRASi as compared with no-PreRASi are shown. *Logrank p*-value is analyzed with using the propensity score matching (adjusted). The inset shows the same data on an enlarged *y* axis

The survival curves with using PreRASi or no-PreRASi are shown in Fig. 3 (unadjusted) and Fig. 4 (adjusted with PSM). The mean, median, and interquartile ranges of the follow-up time were 4.26 years, 3.79 years, and 1.24–6.79 years, respectively. From 1 to 6 year, there was no significant difference in adjusted mortality between PreRASi and no-PreRASi (7.96% vs. 9.79%; RR 0.81, 95% CI 0.74–0.88 and 21.99% vs. 23.00%; RR 0.96, 95% CI 0.89–1.03). Sensitivity analyses performed with the use of the Cox model and the IPW analysis yielded similar results (see Supplementary Information).

### Effects of PostRASi on outcomes

**Fig. 5:**
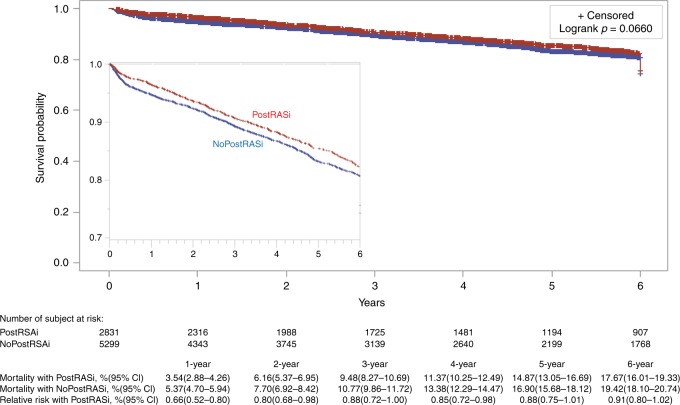
Rates of survival in patients. Cumulative mortality with and without postoperative RASi (PostRASi) and the relative risk (95% confidence intervals in brackets) of PostRASi as compared with no-PostRASi are shown*. Logrank p*-value is analyzed with using the Kaplan–Meier method (unadjusted). The inset shows the same data on an enlarged *y* axis

**Fig. 6:**
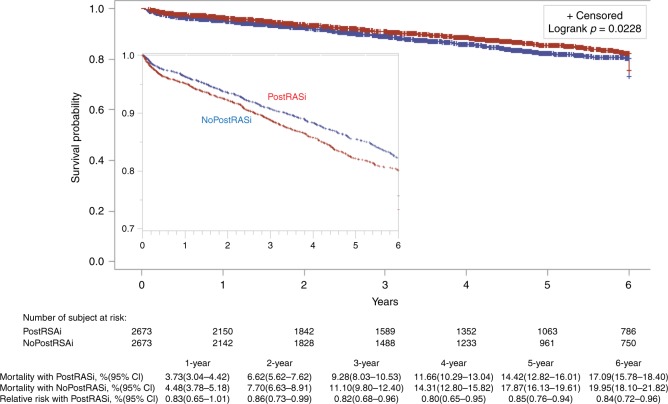
Rates of survival in patients. Cumulative mortality with and without postoperative RASi (PostRASi) and the relative risk (95% confidence intervals in brackets) of PostRASi as compared with no-PostRASi are shown. *Logrank p*-value is analyzed with using the propensity score matching (adjusted). The inset shows the same data on an enlarged *y* axis

The databases of this cohort of patients provided the discharge prescription of RASi but did not have the records of refill or adherence of RASi, thus this study examined the association between postoperative discharge RASi and long-term mortality. Overall, the discharge prescription of RASi was associated with significant beneficial effects on long-term survival. The mean, median, and interquartile ranges of the follow-up time were 4.43 years, 4.03 years, and 1.55–6.90 years, respectively. The unadjusted survival curves trended to show the survival benefit with RASi (Fig. 5) but it did not reach significant differences between with and without postoperative RASi groups at 3, 5 and 6 year (9.48% vs. 10.77%, RR 0.88, 95% CI 0.72–1.00; 14.87% vs. 16.90%, RR 0.88, 95% CI 0.75–1.01; 17.67% vs. 19.42%, RR 0.91, 95% CI 0.80–1.02, respectively). The survival curves adjusted with the use of the PSM are shown in Fig. 6. At 2, 4, and 6 year, there were significant differences in long-term mortality between with and without postoperative RASi groups (6.62% vs. 7.70%, RR 0.86, 95% CI 0.73–0.99; 11.66% vs. 14.31%, RR 0.80, 95% CI 0.65–0.95; 17.09% vs. 19.95%, RR 0.84, 95% CI 0.72–0.96, respectively).

Sensitivity analyses performed with the use of the Cox model and the IPW adjusted analysis yielded similar results (see Supplementary Figs. 1 and 2).

## Discussion

The results of this multicenter and cohort study showed that preoperative RASi therapy (vs. no preoperative RASi) was associated with a significant decrease of 30-day mortality but no significant changes in postoperative MACE or long-term survival. Postoperative RASi therapy, i.e., patients with the prescription of discharge RASi, was associated with a significant decrease in long-term mortality, up to 6 years after cardiac surgery (17.09% vs. 19.95%, RR 0.84, 95% CI 0.72–0.96, *P* = 0.0228), suggesting an association between perioperative RASi and benefits of survival (for both short and long-term survival).

As previous studies showed, ACE inhibitors block the conversion of angiotensin-I to angiotensin-II (a potent vasoconstrictor), and block the breakdown of bradykinin (a potent vasodilator). The effects of angiotensin-II involve all organs and systems in vivo, including cardiovascular, nervous, urinary, endocrine, inflammation, and cell growth^16^. Blockade of RAS by RASi decreases formation of angiotensin II, increases bradykinin, reduces secretion of aldosterone and vasopressin, with decreased activity of the sympathetic nervous system as well as the trophic effects of angiotensin II. Thus, RASi, by influencing extracellular matrix content, cellular apoptosis, tissue inflammation, and fibrosis, could hypothetically reverse the left ventricular-remodeling process, attenuate aortic valve thickening and prevent thrombosis and plaque rapture^6,16,17^.

In the early 2000’s, the QUO VAIDS trial^9^ and APRES trial^10^ reported that in patients undergoing CABG or percutaneous coronary intervention, quinapril, or ramipril, given before or immediately after coronary revascularization for a long-term (>1 year) can decrease MACE including MI, recurrence of angina pectoris, ischemic stroke, transient ischemic attack, clinical heart failure, or need for revascularization. Those two trials were randomized, although had small sample size (*n* = 149 or 159, respectively).

In 2008, the IMAGINE trial^11^(*n* = 2553) tested whether early initiation (7 days after CABG) of an ACE inhibitor reduced cardiovascular events in stable patients with left ventricular ejection fraction 40%. The trial showed that quinapril (40 mg/day, started ≤ 7 days after surgery) did not improve clinical outcomes including cardiovascular events and death up to 3 years after the surgery in low-risk CABG patients. Instead, quinapril, given postoperatively, increased MACE including cardiovascular death, cardiac arrest, nonfatal MI, coronary revascularization, stroke, and unstable angina or heart failure required hospitalization in the early postoperative period (HR 1.52, 95% CI 1.03–2.26, *P* = 0.0356). The results of the IMAGINE trial suggested that ACE inhibitors may cause harm to patients undergoing cardiac surgery. The study has its strength of randomization. Nonetheless, this study may have some limitations such as the inclusion of only “low-risk” patients undergoing CABG, starting quinapril acutely in the postoperative period, and possible excessive doses, as a significantly higher incidence of hypotension was found in the patients receiving quinapril (12%) vs. those receiving placebo (5.5%, *p* < 0.001).

In addition, two large cohort studies reported conflicting results. Based on 3052 patients on ACE inhibitors treatment matched to a control group at single center, Miceli et al.^12^ reported (in 2009) that preoperative therapy with ACE inhibitors was associated with an increased risk of mortality (OR 2.00, 95% CI 1.17–3.42, *P* < 0.03) in patients undergoing CABG. In contrast, based on a prospective cohort study of 4224 patients undergoing CABG from multicenters (72 medical institutions), Drenger et al.^13^ reported (in 2012) that continuation of ACE inhibitors or de novo ACE inhibitors therapy early after CABG was associated with improved inhospital outcomes, including death, cardiac, cerebral, and renal complication. Of note, in the study by Miceli et al.^12^ the dose of ACE inhibitors given in this cohort of patients was likely too high, as evidenced by increased inotropic use in these patients. In the study by Drenger et al.^13^, however, no clinically significant differences in blood pressure and cardiac output were noted among the groups.

Recently RIAS trial (2015) showed that ACE inhibition leads to a modest, but progressive reduction in left ventricular mass in asymptomatic patients with moderate to severe aortic stenosis compared with placebo, with trends towards improvements in myocardial physiology and slower progression of valvular stenosis^18^. In a cohort study (2014), the prescription RASi after surgical aortic valve replacement was found to be associated with improved long-term survival (up to 10 yeas) in patients with severe aortic stenosis (594 matched pairs of treated and untreated patients)^8^. The latest clinical study (2018) showed that among patients who underwent transcatheter aortic valve replacement, receiving a prescription of RASi at hospital discharge compared with no prescription was significantly associated with a lower risk of mortality and heart failure readmission^19^.

With regard to those conflicting results as above, large clinical trials have established beyond reasonable doubt the value of ACE inhibitors for patients with risk of CVD^1–4^. While researchers have known for some time that when a trial demonstrates an overall effect, whether beneficial or harmful, the direction of this effect is usually consistent within subgroups, as described by DeMets and Califf^20^. Thus, questions arise on whether ACE inhibitors are harmful or beneficial to a subgroup of CVD patients: cardiac surgery patients.

As shown in previous studies, hypotension or “vasoplegia” is still a potential side effect of perioperative use of ACE inhibitors^11,21–25^. The history of ACE inhibitors development in patients with acute MI (AMI) is very interesting and may repeat itself in other subsets of patients with CVD^1,2^. In the early 1990s investigators started examining the effects of ACE inhibitor application in patients suffering AMI. An early study, CONSENSUS II trial (in 1992)^26^ showed that enalapril therapy did not improve survival in patients with AMI, instead caused more early hypotension. This disappointing result, nonetheless, was soon overcome by a series of clinical trials that demonstrated the remarkable survival benefits of ACE inhibitors in patients with AMI. A key is that ACE inhibitors were titrated carefully in the immediate postinfarct period in these trials to avoid the development of hypotension^27–30^. In the HOPE trial^1^, a low dose of ramipril (starting with 2.5 mg per day to a full dose of 10 mg per day) or placebo were given to patients for a mean of 5 years. As a result, the risk reduction was largely independent of lowering blood pressure in the ramipril group (only 2–3 mmHg reduction), suggesting that the inhibition of tissue ACE-mediated processes by ramipril therapy without significant hypotensive effect was responsible for the benefits of ramipril.

Overall, careful titration of low-dose RASi, to avoid excessive hypotension, in patients undergoing cardiac surgery may unmask the outcome benefits provided by preoperative RAS inhibitor therapy. Based on the findings from the present study and previous studies, postoperative RASi should be prescribed indefinitely to patients undergoing CABG and/or valve surgery (if no contraindication), particularly due to its long-term survival benefits^1,2,8^. An observational study design, as shown in this study, from a large clinical databases can be more representative to real patient population, may better reflect real-world practice, relatively inexpensive, the ethical issues appear less complex (no patients are randomized). In addition, the studies are in progress to use the big data and/or large observational study for causal analyses, besides correlation analyses^31–33^.

Concerning the limitations of this study, first, although the PSM and other sensitivity analyses were used in this study to reduce overt biases, the potential flaws of a nonrandomized study may remain. Second, clinically it would be appropriate to classify the patients into two groups: taking RASi during both the pre- and postoperative period (continuation) and not taking RASi at any point during the perioperative period (no RASi). However, it would introduce an “immortal” time bias since exposed patients who received their first prescription had to survive until their second prescription^34^. Third, we lacked data on the dose and adherence of RASi in this cohort of patients. The reported rates of patient RASi adherence for cardiovascular protection are high, range from 68 to 92% in the literature^35,36^. Finally, both CABG and valve surgery patients were included in this study since RASi have been shown to be beneficial in both groups of patients^1,2,6–10,12,18,19^. The association we found in this study suggests that perioperative RASi may provide cardiovascular protection with potential long-term benefits for survival. The results of this study also indicate that further investigations, including both RCTs and pragmatic studies are still needed to assess the safety and effectiveness of RASi in patients undergoing cardiac surgery.

## Methods

### Study design and oversight

This study was a multicenter, retrospective, and cohort study involving consecutive patients (*n* = 9584) receiving cardiac surgery including CABG and/or valve surgery at three tertiary medical centers (Thomas Jefferson University Hospital, Abington Memorial Hospital and UC Davis Medical Center, dated from 2001 to 2015). The study was in compliance with the Declaration of Helsinki^37^, approved by Jefferson Institutional Review Boards (IRBs, it covers both Thomas Jefferson University hospital and Abington Memorial Hospital-Jefferson Health) and University California Davis IRB. The individual consent was waived in compliance with the HIPAA regulations and the waiver criteria. After the exclusion, the patients were divided into two groups with or without PreRASi (*n* = 8581), or two groups with or without PostRASi (*n* = 8130), respectively. Preoperative or postoperative use of RASi is defined as any patient who received RASi within 48 h preceding surgery or was prescribed RASi at discharge.

### Data collection

The patient data collected included demographics, patient history, medical record information, preoperative risk factors, preoperative medications, intraoperative, and postoperative data. For missing variables at low-missing rate, the values were imputed to the median for continuous variables (after stratifying on relevant variables to enhance prediction of the missing value), and the most frequent value for binary and polytomous variables. For missing at random, multiple imputation procedures were applied to replace each missing value with a set of plausible values that represent the uncertainty about the right value to impute^38,39^.

### Measurement of outcomes

The primary endpoint was the mortality. The 30 day and long-term all-cause mortality was determined from the Society of Thoracic Surgeons (STS) Registry of the study sites and the Social Security Death Index^40^. The survival time (time-to-event) of the subject began when the subject had cardiac surgery, and ended when the end-point (the death) was reached or the subject was censored from the study^41^. Other outcomes, as defined by the STS national criteria, of this study include postoperative renal failure, readmission, intensive care unit (ICU) length of stay, and a composite outcome—major adverse cardiovascular events (MACE), the latter included permanent or transient stroke, coma, perioperative MI, heart block, and cardiac arrest^42^.

### Adjustment for differences between groups

It was anticipated that patients with or without preoperative or postoperative RASi would differ significantly with respect to baseline (before surgery) characteristics. The propensity scores, reflecting the probability that a patient would receive preoperative RASi, were developed with the use of logistic regression to adjust for between-group differences in baseline characteristics of the patients. The individual variables included in the propensity model are listed in Tables 1 and 3. The PSM was used for estimating treatment effects for specific groups. We matched patients based on the propensity score using a caliper width of 0.2 of the pooled standard deviation of the propensity score, and compare with the results on the basis of greedy matching with 3 or 4 decimal places^43–45^.

### Statistical analysis

Continuous and categorical variables are reported as mean ± SD or percentages, and analyzed by Pearson chi-square test for categorical variables and the Wilcoxon rank-sum test for continuous variables. MACEs were analyzed with using Pearson chi-square test or Wilcoxon rank-sum test (unadjusted) and then PSM (adjusted). For long-term survival, survival curves were estimated with using the Kaplan–Meier method (unadjusted)^46^, then reestimated with using the PSM approach (adjusted). For each group with or without preoperative or postoperative RASi, the survival curves adjusted with the use of the PSM represented the expected rate of survival if the treatment of interest (with or without preoperative and postoperative RASi) were applied to all study patients. Using estimated rates of survival among patients undergoing cardiac surgery with or without preoperative and postoperative RASi, we calculated risk ratios at specific time points and used bootstrap methods to obtain 95% confidence intervals (CI), adjusting for multiplicity and false discovery to assess the effects of RASi for survival for 1–6 year.

To conduct sensitivity analysis, survival curves were reestimated separately for patients with or without preoperative and postoperative RASi with the use of Cox proportional-hazard models without propensity scores^47^. Covariates for each model were identical to those in the propensity model described above. Further, the IPW approach was also used to examine the average treatment effect among the study population^48–50^. In order to limit the influence of extreme IPW, we used truncation of weights by setting the propensity score to the standard range. Percentages, RR, odds ratio (OR), 95% CI, and *P* values (two-sided) < 0.05 were given in the results. SAS version 9.4 (SAS Institute, Inc., Cary, NC) and SPSS 17.0 software for Windows (SPSS Inc., Chicago, IL) were used for the statistical analysis.

### Reporting summary

Further information on research design is available in the Nature Research Reporting Summary linked to this article.

## Supplementary information

Supplementary information

Reporting summary

## Data Availability

All data generated or analyzed in this study are available from the corresponding author on reasonable request.
