# Supplementary material for: Perioperative use of renin-angiotensin system inhibitors and outcomes in patients undergoing cardiac surgery
Source: Nat Commun. 2019 Sep 13;10:4202. doi: 10.1038/s41467-019-11678-9 (PMC6744557; doi:10.1038/s41467-019-11678-9)
Supplement: Supplementary file 1 — Supplementary information [file 41467_2019_11678_MOESM1_ESM.pdf]

Perioperative renin-angiotensin system inhibitors and outcomes in patients undergoing cardiac surgery. A Cohort Study (Supplements)

Qian Ding,[1,2] Zugui Zhang,[3] Hong Liu,[4] Huang Nie,[1,5]

Mark Berguson,[1] Jordan Goldhammer,[1] Nilas Young,[6] Douglas Boyd,[6]

Rohinton Morris,[7] Jianzhong Sun[1]

Author Affiliations: [1]Department of Anesthesiology, [7]Division of Cardiothoracic Surgery, Sidney Kimmel Medical College, Thomas Jefferson University, Philadelphia, PA 19107, USA; Department of anesthesiology, [2] Tangdu Hospital, [5]Xijing Hospital, Xian, P.R. China; [3]Center for Outcomes Research, Christiana Care Health System, Newark, DE, USA [4]Department of Anesthesiology and Pain Medicine, [6]Division of Cardiothoracic Surgery, University of California Davis Medical Center, Sacramento, CA 95817, USA

Supplementary Methods

1. Imputation of Missing Variables and Variables Included in the Propensity Model

It was anticipated that the patient population with and without RASi would differ substantially with respect to preoperative characteristics. We therefore collected baseline variables available in our databases to make adjusted comparisons. Variables common to in the databases were identified from Versions 2.61 and 2.81 of the STS data specifications. Most variables were >99% complete in both groups. Missing values of continuous risk factors were imputed by stratifying on treatment group and combinations of other related risk factors, and imputing stratum-specific medians. This approach was used for weight and height (stratification by gender and treatment group). Categorical variables had <1% missing data and were imputed to the most common category. Although a single imputation approach was used for our primary IPTW analyses, additional analyses were performed using multiple imputation methodology.[1,2] The propensity scores, reflecting the probability that a patient would receive preoperative RASi, were developed with the use of logistic regression to adjust for between-group differences in baseline characteristics of the patients.[3, 4] Covariates in the propensity model were: age, gender, height, BMI, smoking status, family history of coronary artery disease, hypertension, diabetes, cerebrovascular disease, chronic lung disease, peripheral arterial disease, history of congestive heart failure, prior myocardial infarction, angina prior to the procedure, urgent procedure, preoperative medicines including beta-blockers, aspirin, lipid-lowering agents, CABG or valve surgery. Continuous variables were modeled as a flexible polynomial with linear and quadratic components.

2. Sensitivity Analysis

We used several sensitivity analyses to account for possible misspecification of the propensity model, survival curves were re-estimated using a regression based approach that did not utilize propensity scores, i.e., the Cox proportional hazards model with time-varying hazard ratios was used to estimate the association between baseline covariates and subsequent survival separately within with or without RASi cohorts.[5] Covariates for each

model were identical to the propensity model. Using these models, we estimated the average survival curves that would be predicted if all patients in the study were with RASi or if all patients were without RASi perioperatively. Second, the inverse probability weighted (IPW) approach was also used to examine the average treatment effect among the study population.[6-8] For each group with or without preoperative or postoperative RASi, the survival curves adjusted with the use of IPW represent the expected rate of survival if the treatment of interest were applied to all study patients. Risk ratios at specific time points were calculated with estimated rates of survival among patients receiving cardiac surgery with or without preoperative and postoperative RASi, 95% confidence intervals were obtained with bootstrap methods. The characteristics of the patients in the unadjusted, IPTW and PSM groups are shown in Table 1 and 2. Survival curves based on the IPW analysis were found to be nearly identical to ones based on the PSM analysis (Supplementary Fig.1 and 2).

Supplementary References

1. Weintraub WS, Grau-Sepulveda MV, Weiss JM, O'Brien SM, Peterson ED, Kolm P, Zhang Z, Klein LW, Shaw RE, McKay C, Ritzenthaler LL, Popma JJ, Messenger JC, Shahian DM, Grover FL, Mayer JE, Shewan CM, Garratt KN, Moussa ID, Dangas GD, Edwards FH. Comparative Effectiveness of Revascularization Strategies. N Engl J Med 366, 1467-1476 (2012).

2. Shahian DM, O'Brien SM, Sheng S, Grover FL, Mayer JE, Jacobs JP, Weiss JM, Delong ER, Peterson ED, Weintraub WS, Grau-Sepulveda MV, Klein LW, Shaw RE, Garratt KN, Moussa ID, Shewan CM, Dangas GD, Edwards FH. Predictors of long-term survival after coronary artery bypass grafting bypass grafting surgery: results from the Society of Thoracic Surgeons Adult Cardiac Surgery Database (the ASCERT study). Circulation. 125, 1491-500 (2012).

3. Rubin DB. Estimating causal effects from large data sets using propensity scores. Annal of Internal Medicine 127, 757-763 (1997).

4. Kurth T, Walker AM, Glynn RJ, Chan KA, Gaziano JM, Berger K, Robins JM. Results of multivariable logistic regression, propensity matching, propensity adjustment, and propensity-based weighting under conditions of nonuniform effect. Am J Epidemiol 163, 262-270 (2006).

5. Therneau TM, Grambsch PM. Modeling survival data: extending the Cox model. New York: Springer, 2000.

6. Robins JM, Mark SD, Newey WK. Estimating exposure effects by modelling the expectation of exposure conditional on confounders. Biometrics 48, 479–95 (1992).

7. Rotnitzky A, Li L, Li X. A note on overadjustment in inverse probability weighted estimation. Biometrika. 97, 997–1001 (2010).

8. Curtis LH, Hammill BG, Eisenstein EL, Kramer JM, Anstrom KJ. Using inverse probability-weighted estimators in comparative effectiveness analyses with observational databases. Med. Care. 45, Suppl 2:S103-S107 (2007).

Supplementary Figures

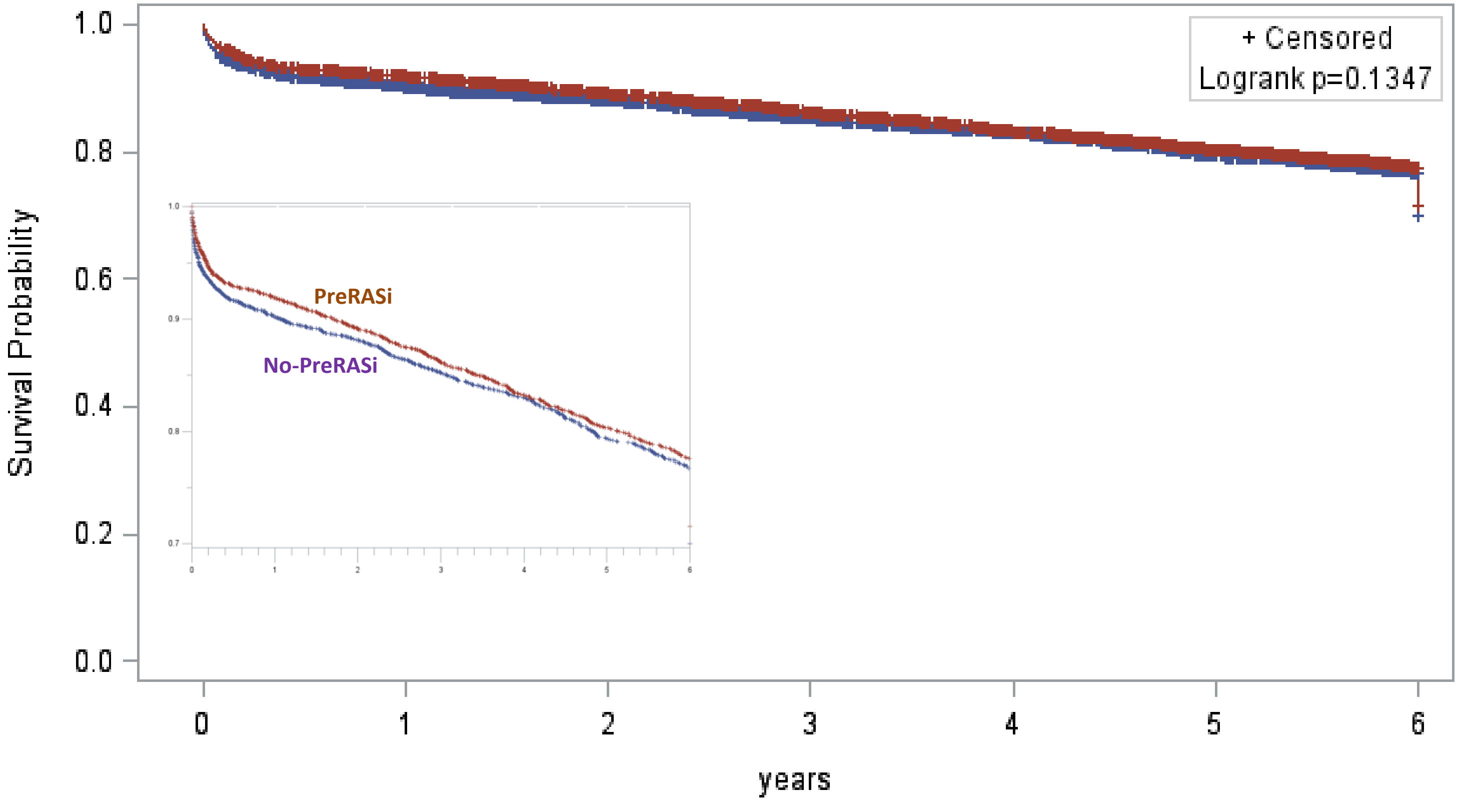

	1-year	2-year	3-year	4-year	5-year	6-year
Mortality with PreRASi %	8.09(7.13-9.05)	10.86(9.74-11.98)	13.80(12.53-15.07)	16.75(15.33-18.17)	19.66(18.10-21.22)	22.37(20.65-24.09)
Mortality with No-PreRASi %	9.81(8.93-10.69)	11.82(10.85-12.79)	14.77(13.66-15.88)	17.01(15.79-18.23)	20.62(19.23-22.01)	23.26(21.75-24.77)
Relative risk with no-PreRASi	0.82(0.75-0.89)	0.92 (0.84-1.00)	0.93(0.86-1.00)	0.98(0.91-1.05)	0.95(0.88-1.02)	0.96 (0.89-1.03)

Fig. 1. Rates of Survival in patients. Cumulative mortality with and without preoperative RASi (PreRASi) and the relative risk (95% confidence intervals in brackets) of PreRASi as compared with no-PreRASi are shown. Logrank p-value is analyzed with using the with the Kaplan-Meier method (unadjusted). The inset shows the same data on an enlarged y axis.

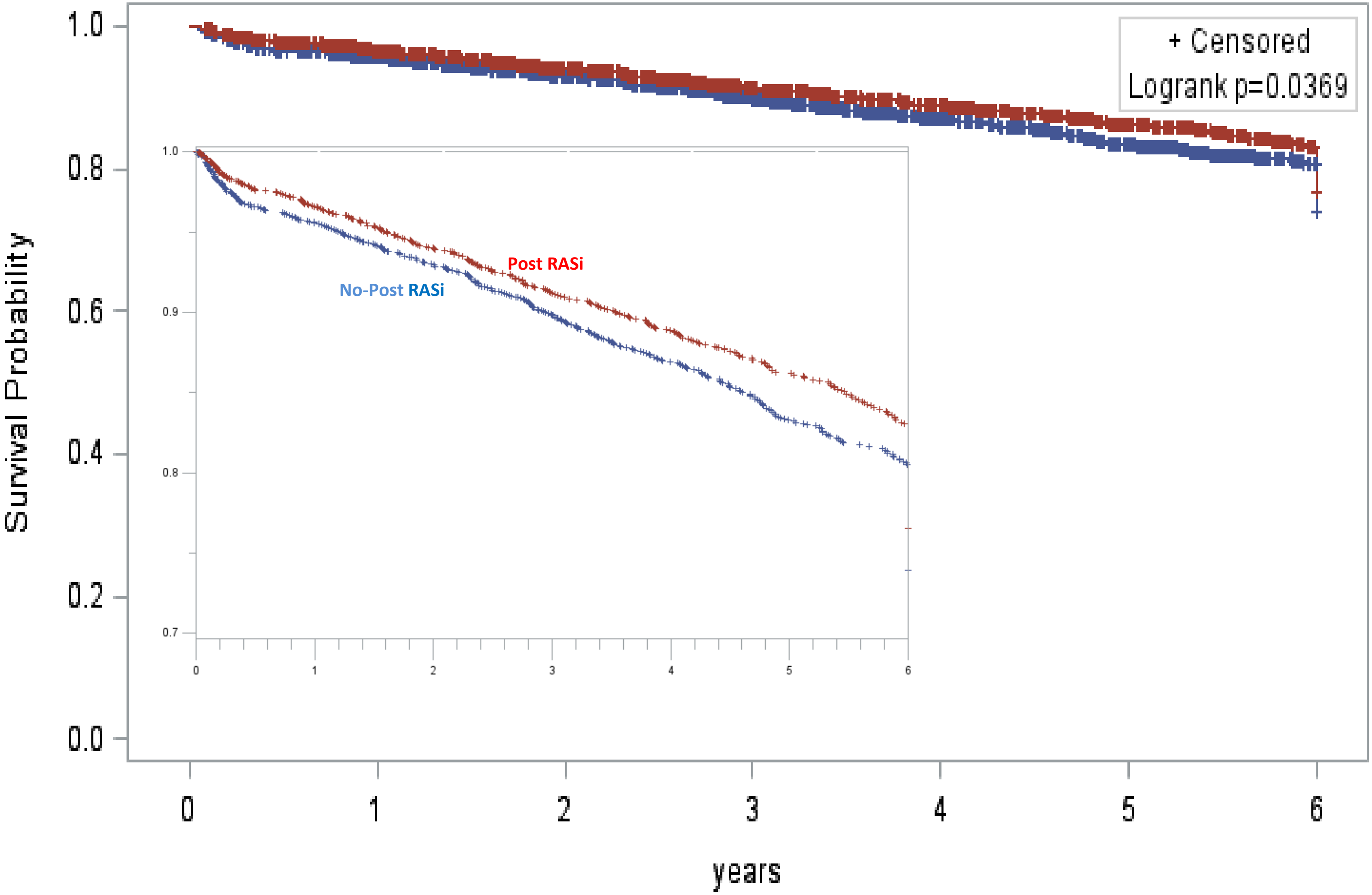

	1-year	2-year	3-year	4-year	5-year	6-year
Mortality with PostRASi %	3.41(2.63-4.19)	6.03(5.02-7.04)	8.84(7.56-10.12)	11.16 (9.72-12.60)	13.79 (12.33-15.25)	17.13 (15.06-19.20)
Mortality with No-PostRASi %	5.17(4.48-5.86)	7.64(6.85-8.43)	10.16(9.92-10.40)	13.13(12.22-14.04)	16.82 (15.24-18.40)	19.4 7(18.21-20.73)
Relative risk with PostRASi	0.66(0.59-0.73)	0.79(0.70-0.88)	0.87(0.80-0.94)	0.85(0.77-0.93)	0.82(0.77-0.87)	0.88(0.78-0.98)

Fig. 2. Rates of Survival in patients. Cumulative mortality with and without postoperative RASi (PostRASi) and the relative risk (95% confidence intervals in brackets) of postoperative RASi (PostRASi) as compared with no-PostRASi are shown. Logrank p-value is analyzed with using the IPW method (adjusted). The inset shows the same data on an enlarged y axis.
