# Supplementary material for: Perioperative use of renin-angiotensin system inhibitors and outcomes in patients undergoing cardiac surgery
Source: Nat Commun. 2019 Sep 13;10:4202. doi: 10.1038/s41467-019-11678-9 (PMC6744557; doi:10.1038/s41467-019-11678-9)
Supplement: Supplementary file 2 — Reporting summary [file 41467_2019_11678_MOESM2_ESM.pdf]

nature research

Corresponding author(s): Jianzhong Sun

Last updated by author(s): Jul 22, 2019

Reporting Summary

Nature Research wishes to improve the reproducibility of the work that we publish. This form provides structure for consistency and transparency in reporting. For further information on Nature Research policies, see Authors & Referees and the Editorial Policy Checklist.

Statistics

For all statistical analyses, confirm that the following items are present in the figure legend, table legend, main text, or Methods section.

n/a	Confirmed	
☐	☒	The exact sample size (*n*) for each experimental group/condition, given as a discrete number and unit of measurement
☐	☒	A statement on whether measurements were taken from distinct samples or whether the same sample was measured repeatedly
☐	☒	The statistical test(s) used AND whether they are one- or two-sided<br>*Only common tests should be described solely by name; describe more complex techniques in the Methods section.*
☐	☒	A description of all covariates tested
☐	☒	A description of any assumptions or corrections, such as tests of normality and adjustment for multiple comparisons
☐	☒	A full description of the statistical parameters including central tendency (e.g. means) or other basic estimates (e.g. regression coefficient) AND variation (e.g. standard deviation) or associated estimates of uncertainty (e.g. confidence intervals)
☐	☒	For null hypothesis testing, the test statistic (e.g. *F*, *t*, *r*) with confidence intervals, effect sizes, degrees of freedom and *P* value noted<br>*Give P values as exact values whenever suitable.*
☒	☐	For Bayesian analysis, information on the choice of priors and Markov chain Monte Carlo settings
☒	☐	For hierarchical and complex designs, identification of the appropriate level for tests and full reporting of outcomes
☒	☐	Estimates of effect sizes (e.g. Cohen's *d*, Pearson's *r*), indicating how they were calculated

Our web collection on statistics for biologists contains articles on many of the points above.

Software and code

Policy information about availability of computer code

Data collection	n/a
Data analysis	n/a

For manuscripts utilizing custom algorithms or software that are central to the research but not yet described in published literature, software must be made available to editors/reviewers. We strongly encourage code deposition in a community repository (e.g. GitHub). See the Nature Research guidelines for submitting code & software for further information.

Data

Policy information about availability of data

All manuscripts must include a data availability statement. This statement should provide the following information, where applicable:

- Accession codes, unique identifiers, or web links for publicly available datasets
- A list of figures that have associated raw data
- A description of any restrictions on data availability

All data generated or analyzed in this study are available from the corresponding author on reasonable request.

Field-specific reporting

Please select the one below that is the best fit for your research. If you are not sure, read the appropriate sections before making your selection.

☒ Life sciences ☐ Behavioural & social sciences ☐ Ecological, evolutionary & environmental sciences

For a reference copy of the document with all sections, see nature.com/documents/nr-reporting-summary-flat.pdf

Life sciences study design

All studies must disclose on these points even when the disclosure is negative.

Sample size	This study was a multicenter, retrospective and cohort study involving consecutive patients (n = 9584) receiving cardiac surgery including CABG and/or valve surgery at three tertiary medical centers. After the exclusion, the patients were divided into 2 groups with or without PreRASi (n =8581), or 2 groups with or without PostRASi (n = 8130) respectively. This is a large clinical study with more than 8000 patients in the study.
Data exclusions	Among total 9584 patients, 1003 patients were excluded from PreRASi groups (fianl n = 8581) and 1454 excluded from PostRASi groups (final n= 8130). The exclusion criteria are given in Fig. 1.
Replication	MACEs were analyzed with using Pearson chi-square test or Wilcoxon rank-sum test (unadjusted) and then propensity scores matching(PSM) (adjusted). For long-term survival, survival curves were estimated with using the Kaplan-Meier method (unadjusted), then re-estimated with using the PSM approach (adjusted). To conduct sensitivity analysis, survival curves were re-estimated separately for patients with or without preoperative and postoperative RASi with the use of Cox proportional-hazard models without propensity scores. Covariates for each model were identical to those in the propensity model described above. Further, the inverse probability of treatment weights (IPTW) approach was also used to examine the average treatment effect among the study population.
Randomization	This study is a multicenter, retrospective and cohort study.
Blinding	The data collectors are blinded for the study assignments and outcomes.

Reporting for specific materials, systems and methods

We require information from authors about some types of materials, experimental systems and methods used in many studies. Here, indicate whether each material, system or method listed is relevant to your study. If you are not sure if a list item applies to your research, read the appropriate section before selecting a response.

Materials & experimental systems

n/a	Involved in the study	
☒	☐	Antibodies
☒	☐	Eukaryotic cell lines
☒	☐	Palaeontology
☒	☐	Animals and other organisms
☒	☐	Human research participants
☐	☒	Clinical data

Methods

n/a	Involved in the study	
☒	☐	ChIP-seq
☒	☐	Flow cytometry
☒	☐	MRI-based neuroimaging

Clinical data

Policy information about clinical studies

All manuscripts should comply with the ICMJE guidelines for publication of clinical research and a completed CONSORT checklist must be included with all submissions.

Clinical trial registration	this study is not a clinical trial.
Study protocol	not a clinical trial.
Data collection	This study is a multicenter, retrospective and cohort study involving consecutive patients (n = 9584) receiving cardiac surgery including CABG and/or valve surgery at three tertiary medical centers (Thomas Jefferson University Hospital, Abington Memorial Hospital and UC Davis Medical Center, dated from 2001 to 2015).
Outcomes	The primary endpoint was the mortality. The 30-day and long-term all-cause mortality was determined from the Society of Thoracic Surgeons (STS) Registry of the study sites and the Social Security Death Index. The survival time (time-to-event) of the subject began when the subject had cardiac surgery, and ended when the end-point (the death) was reached or the subject was censored from the study. Other outcomes, as defined by the STS national criteria, of this study include postoperative renal failure, readmission, intensive care unit (ICU) length of stay and a composite outcome – major adverse cardiovascular events (MACE), the latter included permanent or transient stroke, coma, perioperative MI, heart block and cardiac arrest.
